# Systematic approach towards establishing a National Inventory of Dangerous Pathogens

**DOI:** 10.1080/16549716.2021.1971866

**Published:** 2021-09-08

**Authors:** Iris M. Vennis, Diederik A. Bleijs, Sabrina Brizee, Harold H.J.L. Van Den Berg, Evelien Kampert, Saskia A. Rutjes, Mark W. J. Van Passel

**Affiliations:** a Centre for Infectious Disease Control, Laboratory for Zoonoses and Environmental Microbiology, National Institute for Public Health and the Environment, Bilthoven, The Netherlands; b Biosecurity Office, National Institute for Public Health and the Environment, Bilthoven, The Netherlands; c Ministry of Health, Welfare and Sport, Directorate International Affairs, The Hague, The Netherlands

**Keywords:** Biosafety, biosecurity’, biorisk management, national oversight, material control and accountability, Global Health Security, Biological Weapons Convention (BWC), International Health Regulations (IHR), United Nation Council Security Resolution 1540 (UNCSR 1540)

## Abstract

International regulations stipulate that countries need to organize their biosafety and biosecurity systems to minimize the risk of accidental (biosafety) or malicious intentional (biosecurity) release of dangerous pathogens. International Health Regulations (IHR) benchmarks from the WHO state that even for a level of limited capacity countries need to ‘Identify and document human and animal health facilities that store/maintain dangerous pathogens and toxins in the relevant sectors and health professionals responsible for them’. This study provides a stepwise, systematic approach and best practices for countries to initiate a national inventory of dangerous pathogens. With a national inventory of dangerous pathogens a country can identify and document information in a dedicated electronic database on institutes that store or maintain dangerous pathogens. The systematic approach for the implementation of a national inventory of dangerous pathogens consists of four stages; identification, preparation, implementation, and maintenance and evaluation. In the identification phase, commitment of the relevant national ministries is to be established, and a responsible government entity needs to be identified. In the preparatory phase, a list of pathogens to be incorporated in the inventory, as well as a list of institutes to include, is to be agreed upon. In the implementation phase, the institutes are contacted, and the collected data is stored safely and securely in a electronical database. Finally, in the maintenance and evaluation phase meaningful insights are derived and reported to the relevant government authorities. Also, preparations for updates and modifications are undertaken, such as modifications of pathogen lists or institute lists. The approach and database, which is available from the authors, have been tested for the implementation of a national inventory of dangerous pathogens in multiple East-African countries. A national inventory of dangerous pathogens helps countries in strengthening national biosafety and biosecurity as well as in their compliance to IHR.

## Background

Within the frameworks of international regulations and treaties, signatory parties are urged to account for dangerous pathogens in their countries. The multilateral Biological Weapons Convention (BWC) was established in 1972 to ban the development, production and stockpiling of biological weapons of mass destruction [1]. Signatory countries agreed to implement measures in order to improve international co-operation in the field of peaceful biological activities. In addition, the G7 Global Partnership against the spread of weapons and materials of mass destruction emphasises the importance to ‘*secure and account for materials that represent biological proliferation risks*’ [2]. Moreover, the United Nation Council Security Resolution 1540 (UNCSR 1540) of 2004 with the obligation to ‘*Develop and maintain appropriate effective measures to account for and secure such items* (i.e. nuclear, chemical or biological weapons) *in production, use, storage or transport*’ [3] was adopted unanimously. Signatory parties are urged to take appropriate measures to secure and account for materials that represent biological proliferation risks. However, these regulations and treaties provide limited guidance for policymakers on what particular pathogens should be accounted for, or how an accountability and regulatory system should or could be set up. The World Health Organisation Joint External Evaluations (WHO JEE) acknowledge that the lack of accounting systems in many countries is a significant biosecurity gap [4]. In order to address this biosecurity gap and to prevent misuse of dangerous pathogens, governments would first need to gain a comprehensive understanding on which dangerous pathogens and related materials are in fact present in their country.

Despite growing risks, such as disease outbreaks [5] or intentional biological attacks [6], most countries do not have the required capabilities to prevent, detect, and respond to high consequence biological events [7]. This has become apparent when analysing the outcomes of the Joint External Evaluations that have been completed by 110 countries as of 8 May 2020. In total, 48 indicators from 19 technical areas have now been assessed for each of the 96 countries that have published their report on WHOs website [8]. In 2018, countries’ average results reveal that 43% of the total number of all assessed indicators showed limited or no broad-based health security capability, and very low capability is observed for specific indicators focused on biosecurity, biosafety, and the ability to link public health with security authorities [4,9]. These findings are instantiated by the current COVID-19 pandemic, with many countries displaying a limited capacity to prevent, detect, and respond to this high consequence biological event. From the numerous WHO JEEs that have been conducted, it became apparent that one of the most frequent biosafety and biosecurity recommendation is that countries need to set up and implement a national pathogen inventory system in order to regulate and control pathogens that are most vulnerable for misuse and theft. The fact that numerous countries still lack a meaningful system to account for institutes storing and maintaining dangerous pathogens indicates that such inventories constitute a significant biosecurity gap worldwide.

A critical step towards greater global health security and encouraging safe and secure practises in biological laboratories starts with a national oversight system to address the biological risks and mitigation measures associated with handling and storing dangerous pathogens. A *‘national inventory of dangerous pathogens’ (NIDP)* is one feasible and practical implementation option, and an essential step, to build a national oversight and regulatory system. Within the context of biosecurity, a national inventory is an electronic database intended to store information collected from all institutes storing and maintaining dangerous pathogens in a coordinated secured location [10]. In this way, the national authorities have access to information on what dangerous pathogens are present in their country and where they are stored. Notably, this inventory is distinctly different from an institute-specific pathogen inventory system, such as the Pathogen Asset Control System (PACS) [11]. The national inventory preferably operates at a national level, and can be a single electronic database that is hosted at one central location. In that case, the database will store information collected from all institutions that maintain dangerous pathogens in one secure database. The primary purpose of the national inventory is to ensure that national authorities can generate an overview of all laboratories that store dangerous pathogens, which in turn could serve as a fundamental foundation to monitor the safety and security practices in biological laboratories throughout the country.

As there is little guidance on systematically establishing a centralized data collection system, this article aims at closing this knowledge gap and provides tools and software for implementing a national database. It supports national authorities and policymakers by providing a stepwise approach and a set of best practises, which are based on previous implementation efforts in different East-African countries, including Uganda [10]. Here, we describe the different phases of implementation and provide guidance on challenges that could be encountered. This includes both practical steps, stakeholders involved in the process, the NIDP database software, and considerations for information and communication security.

## Methods

**Figure 1.**
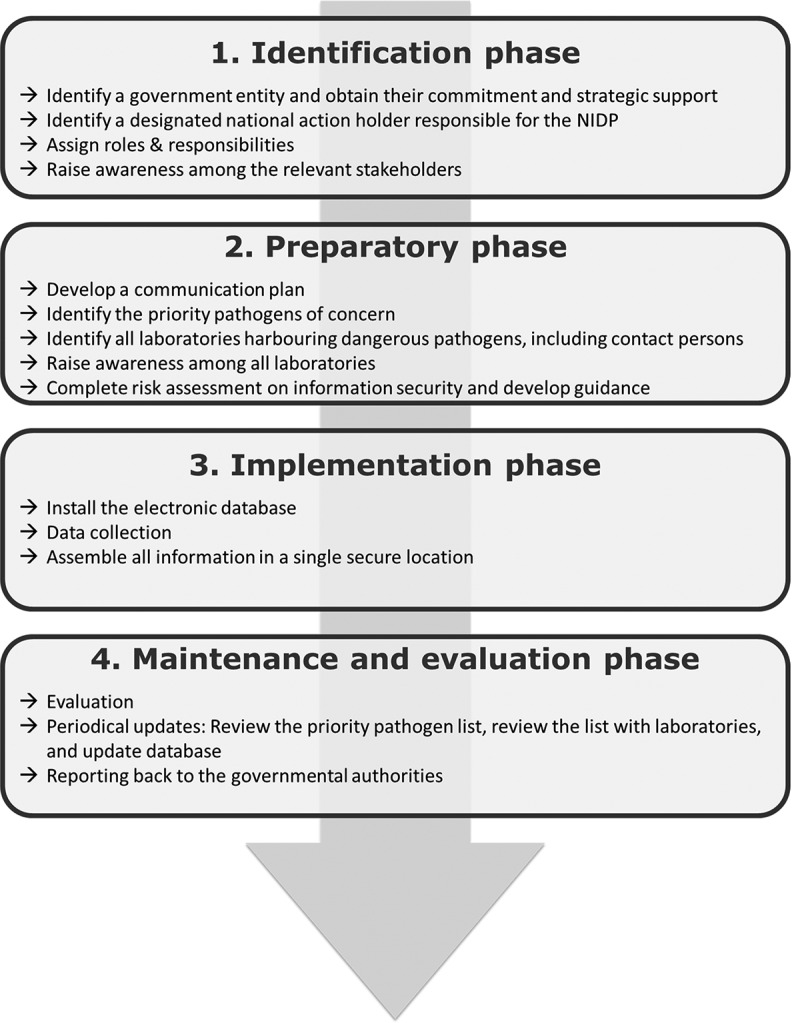
Schematic overview of the different phases and related actions that are required to establish a National Inventory of Dangerous Pathogens

A systematic approach on how to set up a National Inventory of Dangerous Pathogens is suggested. It introduces the four stages on how to embark on the inventory process: 1) the identification phase; 2) the preparatory phase; 3) the implementation phase; and 4) the maintenance and evaluation phase (Figure 1).

### Identification phase

The first step in establishing a national inventory is to raise awareness among stakeholders and determine which government entity will be responsible for overseeing and tracking progress. This government entity needs to make an inquiry to determine which other ministries should be involved in the decision-making process. Next, the governmental entity or entities need to assign an appropriate focal point who interacts with all relevant laboratories, collects the data, maintains the database, and reports back to the responsible government entity.

### Preparatory phase

An essential part of the inventory is to establish a list of dangerous pathogens. This list consists of pathogens that pose a significant biosafety and biosecurity risk within a particular country. Additionally, a pathogen list forms the basis of nearly all other biosecurity regulations, which inevitably means that institutes that handle and store these dangerous pathogens for more than 30 days [12] should integrate appropriate and adequate safety and security practices in their biological laboratories. Subsequently, there has to be decided on an effective database and the elements to be included in the database. The database can be either self-developed or off-the-shelf. As there was no database or software dedicated to national inventory control freely available, the Dutch National Institute for Public Health and the Environment (RIVM) developed a secure, electronic NIDP database intended for national inventory control of dangerous pathogens to be hosted at a central location. RIVM has made this software available on request to allow countries to create and manage a national inventory. The next step is to identify all institutes in the country that store dangerous pathogens. In this context ‘institutes’ refers, but is not limited, to academia, public health, veterinary, agricultural (diagnostic) facilities, foreign-supported research facilities, commercial production or diagnostic facilities and hospitals. Once these institutes are identified and listed, a communication plan is developed to raise awareness and establish formal communication with representatives from each institute.

### Implementation phase

In the implementation phase data is collected and stored in a coordinated secure electronic database that is hosted at a central location. The first step is to determine the software for the database, which should be user-friendly and suitable for all stakeholders working with it. The software has to be installed in consultation with Information Technology (IT) experts and access rights should be granted to authorised persons only. The next step is data collection, the process of gathering information of all invited institutes that handle and store dangerous pathogens, in order to gain a better understanding of the biosecurity situation in a given country. Institutes are requested to submit information on dangerous pathogens’ characteristics, the institute and the responsible persons. Subsequently, once institutes submit the requested information, it can be imported into the database. For security purposes, a secure centralized database may be preferable to the sharing of database copies with all institutes. However, national guidelines on the securely storing of sensitive information should be followed.

### Maintenance and evaluation phase

The maintenance and evaluation phase starts once data is collected and stored in a database. It is an important aspect as it sets a framework to sustainably implement and maintain the national inventory. A data analysis should be performed in order to derive meaningful insights, such as overviews of where certain pathogens are stored or where (information) safety and security considerations may be prioritized. The focal point shall ensure that the outcomes of the data collection and analysis are reported back to the relevant national authorities. As the basic principle of inventory control is to know where dangerous pathogens are stored within the country, periodical updates and customization of the national inventory are strongly recommended to ensure an accurate representation of the national situation. Updates of the database include several aspects, including the software, persons who have access to the database, closed and new institutes, the list of pathogens, and stored data. Maintenance of the database is a continuous process as to ensure all data is up-to-date and available.

## Results & discussion

### Timeframe

The implementation of a national inventory, though procedurally straightforward, could take a considerable amount of time, for example due to the interdepartmental nature of the process, consensus on the responsible governmental entity/owner, the sensitivity of the information, and cybersecurity considerations. The implementation time is dependent on a range of factors, including governance structure, involved stakeholders, and pre-existence of a list of dangerous pathogens. Although there is no set timeframe in which the national inventory can be set up, there are several factors that could foster the implementation process: available resources, financial means, appropriate mandate, and high-level commitment. A feasible timeline for implementing a national inventory is estimated at two years, taking into account that several processes can be run in parallel. The maintenance and evaluation phase will continue indefinitely after implementation. In case early consensus on the division of roles and tasks is reached, the setting up, introduction, implementation and data collection process could be accomplished in less than a year, as demonstrated by the implementation of a National Inventory of Dangerous Pathogens in Uganda [10]. However, the process will be unique in every country and the preparatory and implementation phase could be time consuming.

### Identification phase

The primary purpose of the national inventory is to ensure that national authorities have at their disposal, and can generate a current overview of all laboratories that store dangerous pathogens. In the identification phase, the main challenge is to involve ministries to commit to implementation of an inventory, as it could serve as a fundamental foundation for the government to keep biosecurity oversight and form the basis for national biosecurity legislation. The national inventory fits within the bigger picture of monitoring safety and security practices in biological laboratories throughout the entire country, for which a biosecurity checklist is available [13]. It is key to include collective and high-level commitment, because high-level commitment from the national government could ensure that objectives are met, adequate budget and resources are allocated, and the inventory process is successfully integrated at the national level. In addition, high-level support could encourage laboratories to comply with the request of sharing information on a list of dangerous pathogens stored within their laboratories. Commitment from ministries from different disciplines needs to be achieved, as to promote a one health approach and to connect safety and security. This includes ministries responsible for health security, which address compliance to the Biological Weapons Convention (BWC), and United Nation Council Security Resolution 1540 (UNCSR 1540), and ministries responsible for health safety, which address health risks at the animal-, environment-, and human interfaces.

It is important to reach early consensus on the division of roles and tasks, and appoint a focal point to be the national action holder. The government entity responsible for overseeing and tracking progress depends on national factors, such as the regulatory framework. For example, the national focal point in Uganda was initially the Ministry of Health, but was later assigned to the Uganda National Council for Science and Technology [10]. The focal point should be provided with an appropriate mandate in order to form a team with different professionals, such as a coordinator, an IT expert or biosafety/biosecurity specialist to effectively implement, update, and maintain the national inventory. In addition, the role of the national focal point is to allocate responsibilities and roles within the team to ensure that expectations are set throughout the implementation process. Throughout the entire implementation process, the communication, ownership, and data-collection activities should lie with the national authorities, as to ensure sustainability.

During this phase, both the responsible government entity and national focal point already need to raise awareness on the database among the relevant stakeholders in preparation for the next phase. The relevant stakeholders and their function in the inventory implementation process are displayed in Table 1.

**Table 1.** Stakeholders and their function in the National Inventory of Dangerous Pathogens implementation process

Stakeholders	Contribution
High level representative(s) from governmental body	Adequate resources, authority, mandate. E.g. a representative of Prime Minister’s Office.
Representative(s) from ministries responsible for health security	Expertise in (inter)national biosecurity initiatives and national representative of Biological Weapons Convention (BWC), United Nation Council Security Resolution 1540 (UNCSR 1540), Global Partnership Against the Spread of Weapons and Materials of Mass Destruction, Global Health Security Agenda (GHSA). E.g. representative of Ministry of Defense, Ministry of Foreign Affairs, and/or Ministry of Security
Representative(s) from ministries responsible for health safety	Expertise in (inter)national health safety initiatives on animal-, environment-, and human level and national representative of WHO. E.g. representative of Ministry of Health or Ministry of Agriculture
High level representative(s) from (public) health institute	Expertise of national health situation, health authority, advise on all determinants of health
Top management from (public health) institutes, working with high-risk biological agents	General information on pathogens institutes work with and agreement to share data
Contact points from (public health) institutes, working with high-risk biological agents	Information on pathogens that are present in the country and on the current situation. E.g. from hospitals, laboratories, universities
Biorisk officers/managers	Expertise in biosafety and biosecurity management and on reducing risks posed by pathogens
(Medical) Microbiologists (animal, plant and human)	Expertise of pathogens and the corresponding human, animal, and plant diseases
Database experts	Knowledge and skills of setting up and maintaining an electronic database
IT security specialists	Knowledge and skills to securely implement a database
Communication experts	Expertise in communication, awareness raising and stakeholder engagement

### Preparatory phase

The preparation of a national pathogen list is one of the main activities in this phase. What biological agents should be included on the list requires a great deal of thought and can be quite a challenge. Experts can develop a risk-based list of dangerous pathogens by means of carrying out a systematic risk-based assessment [14] tailored to the national situation. Experts have compiled a combined list of biological agents [15] that could serve as a resource to create a national pathogen list. Alternatively, a country may choose to utilize an international and acknowledged priority pathogen list, for example the Australia Group Expert Control List [16] or the U.S. Department of Agriculture (USDA) and Health and Human Services (HSS) Select Agents and Toxins list [17].

Subsequently, a suitable database and elements to be included need to be decided upon. One can decide to use an existing database or to design a new database, tailored to the needs of a national inventory. Elements regarding information on the institute, pathogen and contact person are proven to be crucial, as they contribute to securing and accounting for dangerous pathogens. Table A1 displays the elements that are standard included in the NIDP database developed by the authors. This list of elements is neither exhaustive nor comprehensive. As elements to be requested can change over time and different elements may be relevant for specific national situations, the NIDP database offers the possibility to add new elements. Because the database will contain sensitive information, the establishment of a database requires a secure IT infrastructure, which needs to comply with national government procedures concerning sensitive information.

Identifying all relevant institutes and establishing communication with these institutes is key in the preparation of a national inventory, in order to create awareness and achieve a complete national overview. Relevant institutes include universities, public health, veterinary, agricultural (diagnostic) facilities, foreign-supported research facilities, commercial production or diagnostic facilities and hospitals. There are different ways to identify all these institutes including the use of web search, existing national laboratory networks, laboratory mapping tools, personal communication, and existing legislative and regulatory systems. Once the institutes are identified, an accurate list with the appropriate points of contact per institute is crucial to ensure that the communication is not being left unnoticed or lost. The focal point will have to develop a proper communication strategy; both for internal meetings, as well as how to communicate with external stakeholders. A communication plan assists in setting standards for how, when, and with whom communication takes place. Moreover, it enables the team to maintain control of the process and ensures that all stakeholders receive all necessary and relevant information.

The final challenge of this phase is to establish commitment from the institutes. A cooperative relation with the institutes is vital to the success of not only the NIPD, but also other biosecurity initiatives. The national authorities need to play a major role in awareness raising activities towards the relevant institutes concerning the concept of the national inventory, the primary purpose of a national inventory, the relevance of such an inventory as a national biosecurity asset, how the national inventory complies with international regulations, as well as explaining what is expected from institutes concerning data sharing. The implementation of the national inventory serves a common interest by preventing security breaches, biological warfare and bioterrorism. In addition, government commitment could also contribute to more compliance to the request of sharing information on dangerous pathogens stored within national laboratories. Activities proven to advance stakeholder participation include stakeholder consultation, awareness workshops, first-run implementation and stakeholder evaluation.

### Cyber security

Security aspects of the database form a major challenge and cybersecurity should be well considered before implementation of a national inventory. This starts with deciding on the software. This can be existing software or self-developed software that complies with national security procedures and is tailored to the national situation. The authors developed the NIDP database, which is a secure, centralized electronic database intended to store information collected from those institutes that handle and store dangerous pathogens. This secure software is available on request to allow countries to create and manage a national inventory. The ownership of the database lies with the authorities using the software, meaning that no sensitive information will be shared with, stored, handled by, or transported to RIVM or other external partners. In addition, the source code is available upon request in order for countries to evaluate it before use or to tailor the database to national needs. For security reasons, it is recommended to save the electronic database at a central, stand-alone location, without connection to the internet.

The instalment of the software is considered a technical aspect of the implementation process, it is recommended to consult IT experts to ensure that the software is installed correctly, securely and accordingly to national security procedures. The IT expert could provide assistance with regards to the organization’s IT capacity, the IT system and the information security policies. Together with the IT expert, the security risks and vulnerabilities within the organization should be identified and addressed. This could include gauging whether the data needs to be encrypted, considering whether it is necessary to provide a layered digital structure for information security, and ensuring that a back-up of the database is available and stored securely. A secure back-up procedure should be in place, in alignment with national protocols concerning databases with sensitive information. Make sure that the importation of the data into the database is done securely. Furthermore a strategy for secure data collection and software updates needs to be developed. This to safeguard that the consolidated national data will not be accessible and available to unauthorized persons. Hence, it is essential to identify and map all risks and seek appropriate measures to mitigate those risks.

### Implementation phase

Once the software is securely installed, information from all invited institutes needs to be collected. The NIDP database subscribes that data can be collected through a dedicated Excel spreadsheet, and subsequently be imported and securely stored into an electronic database. As the data collection is survey driven, it is vital that the purpose of the inventory program and the instructions are well explained to the respondents. Without clear explanations or instructions, the invitation to share (potentially sensitive) information will most probably result in a low response rate. Therefore, the focal point should take the appropriate mandate and security of the data collection process into consideration before inviting institutes to share their information. National protocol should be followed concerning the communication of sensitive information via internet. In case online correspondence presents vulnerabilities, physical collection of data (e.g. via USB memory stick) should be considered. The invitation should be sent out to the top management of the institute to agree on sharing information and appoint an appropriate point of contact within the institute. Furthermore, the response must be managed and assistance needs to be provided to institutes that have specific questions. Once institutes submit their requested data files, it has to be imported into the database.

### Maintenance and evaluation phase

After collecting all the data, novel insights can be derived from the data from the different institutes, such as overviews on what dangerous pathogens are stored in which institutes, how many institutes store dangerous pathogens or the diversity of dangerous pathogens that is present in the country, and possibly recommendations from these institutes on novel infectious disease samples. Based on these insights a country can consider if it is preferable that certain institutes work with specific (types of) dangerous pathogens. Considering safety and security, the government may decide on minimizing the number of institutes that store certain pathogens for more than 30 days, appoint reference laboratories and expertise centres, or improve permit and safety regulations and inspection. The NIDP database stores information per specific year to allow users to analyse the data retrospectively in order to identify changes over time. Currently, the available software as developed by the authors solely allows the data to be analysed manually. However, data can be exported and edited for presentation in charts or graphs. In deriving these insights, it is important to consider what information should be reported to the different stakeholders and which secure communication channels should be used to do so. Information sharing needs to be done in compliance to country-specific procedures and official information confidentiality laws.

Periodically (e.g. annually), completely updated copies of all datasheets from the different institutes should be sent to the national focal point to gain an up-to-date overview of the current biosecurity situation. The communication plan should contain a strategy to actively receive updates from institutes when a situation changes and for periodically requesting institutes to send in updated copies of all datasheets. Biosecurity involves challenges that are continuously changing and evolving, primarily due to newly emerging biological agents, bioterrorist threats, or cutting-edge technologies. Therefore, the national focal point should plan for periodical updates on the pathogen list, the database itself, and backups of the sensitive information. Also the list with institutes should be updated regularly, as some institutes will reorganise or close and new institutes could be established over time. For the principal of continuous improvement, it is critical to periodically evaluate whether the intended outcomes were achieved and any points for improvement can be identified. Therefore, the national focal point should be interested in finding out how the process could be more efficient or streamlined in the future.

### Security considerations

For safe and secure implementation of a national inventory it is important to pay attention to certain security considerations. Physical security measures would prevent unauthorized physical access and damage to or interference with information. The focal point could define various layers of security to protect the specific area in which the computer with the running database is stored, such as card readers, PIN code access, intrusion detection, or personal systems such as biometric systems. In addition, it is key to apply information classification and use a level of confidentiality comparable to other classified information. This also means that information or data could only be accessible to authorized people. The level of confidentiality should be determined in accordance with existing (institutional) procedures and relevant official information confidentiality laws. The assigned classification then prescribes that certain measures are to be taken to restrict access to the information. The importance of handling sensitive information should not be underestimated, as incorrect handling of information could facilitate unauthorized persons to gain access to the national inventory and data. In addition, the output of the national inventory might need to be shared with stakeholders, such as the relevant ministries. Information security policy should therefore not only describe secure procedures for information exchange within the organization but for third parties as well. Potential security measures could include conducting a security screening for people with access to the national inventory and ensuring that employees can anonymously report or share integrity issues. As a coordinated database contains a vast amount of sensitive information, it is of utmost importance that security of the national inventory is guaranteed, similar to other sensitive national databases.

### Lessons learned

This systematic approach towards establishing a National Inventory of Dangerous Pathogens was applied in East-African countries, including Uganda [10], which established a national inventory, and has been presented pending implementation in Ethiopia, Kenya, and Tanzania. In assisting different countries with the implementation of the inventory, several lessons were learned. We experienced the absolute need to designate a government entity responsible for overseeing and tracking progress on the implementation of a national inventory. This governmental focal point has to determine which other ministries should be involved in the decision-making process and needs to assign an appropriate focal point who interacts with all relevant laboratories, collects the data, maintains the database, and reports back to the responsible government entity or entities. High-level commitment from the national government encourages laboratories to share information on dangerous pathogens stored within their laboratories for the purpose of a national inventory.

In addition, to ensure sustainable implementation, we experienced that commitment of countries to improve their national health security and national ownership of the activities included in the process are extremely relevant. The former could be shown by their IHR self-assessments and subsequent public WHO Joint External Evaluations (JEE), and the national drive to address the identified needs and gaps as described in a national action plan. The latter can be achieved by active participation of national partners in all subsequent activities within the process.

To further enhance sustainable implementation and increase biosecurity, the national inventory should be placed in a broader biosecurity scope. An implemented inventory provides an overview of the institutes working with dangerous pathogens. This offers the possibility for regional, national or local authorities to develop, modernize, implement and maintain national biosafety and biosecurity systems. For example *An Analytical Approach: biosafety and biosecurity oversight framework* [18] provides a methodology for such a system. This system can be linked to a system to monitor and assess biosecurity within institutes with tools such as a *biosecurity checklist* [13]. Furthermore, there are multiple tools to assess if institutes working with dangerous pathogens have implemented biosafety and biosecurity measures. For example the *WHO IHR self-assessment tool* [19] and *Joint External Evaluation* [20] offer an analysis on a national level, whereas the *biosecurity vulnerability scan* [21] and the *biosecurity self-assessment toolkit* [22] can be applied on an institutional level. Together they can form a strong foundation for sustainable biosecurity and compliance to the Biological Weapons Convention (BWC), United Nation Council Security Resolution 1540 (UNCSR 1540), and IHR.

## Conclusion

In order for countries to account for dangerous pathogens to comply with international biosecurity regulations, it is important to be aware of which dangerous pathogens are present in the country. A National Inventory of Dangerous Pathogens is often one of the first steps to identify which laboratories in the country store dangerous pathogens. It does not only provide an overview of where these pathogens are located, but also insight into the variety of pathogens that are stored within the country. This article provides guidance on implementing a national inventory, including best practises, tools and dedicated software for a national database. Having a comprehensive overview enables a government to facilitate the identification, holding, and monitoring of dangerous pathogens in their country. A national database could bring national authorities one-step closer to set up a national regulatory and accounting system to encourage safe and secure practises in biological laboratories. This practical tool enables countries to take further steps in securing and accounting for dangerous pathogens, as stipulated by the Biological Weapons Convention (BWC), United Nation Council Security Resolution 1540 (UNCSR 1540), and IHR.

## Data Availability

Please contact the corresponding author to request the electronic NIDP database software.

## Paper context

International regulations request that countries account for and minimize the risk of accidental or intentional release of dangerous pathogens. A centralized data collection system assists countries to account for dangerous biological material and strengthens biosafety and biosecurity capacities. As there is little guidance on systematically establishing such a system, this article provides best practices to initiate the first steps for a national inventory of dangerous pathogens (NIDP) and offers a dedicated electronic database.

## Appendix 1

**Table A1.** Relevant elements that could be included into the electronic database

Field entry	Field options	Explanation
Contact information institute		
Name institute	[*Text input*]	Name of the legal entity of the respective institute
Establishment	[*Text input*]	Legal name of the establishment that is part of the legal entity
Type of institute	[*Checkbox*] Hospital University Public health institute Veterinary institute Agricultural institute Pharmaceutical company Research institute Food authority Other [*text input*]	An organization, institute, or company devoted to the promotion of a particular cause or program
Primary task	[*Checkbox*] Diagnostics Medical research Veterinary research Agricultural research Biotechnology Pharmaceutical development Public health Other [*text input*]	The type of endeavour or task that is pursued within the respective organisation, institute, or company
Address	Street name and number	
	Zip/ postal code	
	P.O. Box	
	City	
	Region	
	Coordinates	
	Telephone	
	Email	
Information biological agent		
Name biological agent	[*Dropdown menu*]	Scientific name of the respective biological agent. For example *Bacillus anthracis*.
Type of biological agent	[*Checkbox*] Bacterium Virus Fungus Parasite Toxin	Describes the type of the respective biological agent
Mode of Transmission	[*Checkbox*] Airborne Oral Faecal Sexual Vector-born Blood-born Parental (mother to child)	A method of transmission is the movement or the transmission of pathogens from a reservoir to a susceptible host. Once a pathogen has exited the reservoir, it needs a mode of transmission to the host through a portal of entry. Transmission can be by direct or indirect contact or through airborne transmission.
Host range	[*Checkbox*] Human Animal Plant Unknown	The host range is the collection of hosts that an organism can use as a partner
Reservoir	[*Checkbox*] Human reservoir Animal reservoir Environmental reservoir Unknown	Any person, animal, plant, soil or substance in which an infectious agent normally lives and multiplies. The reservoir typically harbours the infectious agent without injury to itself and serves as a source from which other individuals can be infected. The infectious agent primarily depends on the reservoir for its survival. It is from the reservoir that the infectious substance is transmitted to a human or another susceptible host.
Risk classification	[*Checkbox*] 2 3 4	Biological agents that are known to infect humans are classified according to Risk Groups (RG), with RG1 as the lowest/least harmful and RG4 as the highest. These pathogens are classified into four hazard groups according to the following criteria: Ability to cause infection; Severity of the disease; Risk that the infection will spread to the population; Availability of vaccines and effective treatment
Contact details person 1 (e.g. CEO of the institute) The Chief Executive Officer (CEO) of the respective institute. The CEO is the highest-ranking executive within the institute, and their primary responsibilities include making major corporate decisions, managing the overall operations and resources of the institute, and acting as the main point of communication between the board of directors and corporate operations.		
Full Name	Title	
	First name	
	Last name	
Contact	Telephone	
	Mobile phone	
	Email	
Contact details person 2 (e.g. Management) A team of individuals at the highest level of management within an organization and has the day-to-day tasks of managing the institute or organisation		
Full name	Title	
	First name	
	Last name	
Position	[*Checkbox*] Head of the department Other [*text input*]	
Contact	Telephone	
	Mobile phone	
	Email	
Contact details person 3 (e.g. Responsible for pathogen) The person within the respective institute who has the authority and the duty of taking care of the particular biological agent		
Full name	Title	
	First name	
	Last name	
Position	[*Checkbox*] Head of the department Biosafety officer Analyst Other [*text input*]	
Contact	Telephone	
	Mobile phone	
	Email	
